# Evaluation of AI-generated exercise prescriptions for diverse cardiac conditions in rehabilitation: a simulation study using the DeepSeek model

**DOI:** 10.3389/fresc.2026.1844420

**Published:** 2026-08-18

**Authors:** Luca Perrero, Menada Gardalini, Tatiana Bolgeo, Patrizia Valorio, Calogero Malfitano, Valter De Michelis

**Affiliations:** 1 Neurorehabilitation Unit, IRCCS AOU SS Antonio e Biagio e Cesare Arrigo, Alessandria, Italy; 2 Department of Research and Innovation (DAIRI), IRCCS AOU SS Antonio e Biagio e Cesare Arrigo, Alessandria, Italy; 3 Psychology Unit, IRCCS AOU SS Antonio e Biagio e Cesare Arrigo, Alessandria, Italy; 4 Department of Biomedical Sciences for Health, University of Milan, Milan, Italy; 5 Azienda di Servizi Alla Persona Istituti Milanesi Martinitt e Stelline e Pio Albergo Trivulzio, Milan, Italy; 6 Cardiac Rehabilitation Unit, IRCCS AOU SS Antonio e Biagio e Cesare Arrigo, Alessandria, Italy

**Keywords:** artificial intelligence, cardiac rehabilitation, clinical decision support systems, exercise therapy, large language models, precision medicine

## Abstract

**Background:**

Cardiac rehabilitation (CR) significantly improves outcomes for patients with cardiovascular disease, but delivering consistently personalized exercise prescriptions remains challenging due to resource limitations and practice variability. Artificial intelligence (AI), particularly large language models (LLMs), may support the generation of guideline-adherent baseline exercise programs.

**Objective:**

This study aimed to assess the adherence to guidelines, expert-evaluated safety, and clinical plausibility of AI-generated exercise prescriptions for cardiac rehabilitation across common cardiac conditions in simulated clinical scenarios.

**Methods:**

Exercise prescriptions generated by DeepSeek were evaluated using five purpose-designed simulated clinical profiles: recent myocardial infarction (<1 month), prior myocardial infarction (>1 month), atrial fibrillation, heart failure with reduced ejection fraction (EF 35%), and heart failure with preserved ejection fraction (EF 50%). For each profile, the model was prompted to generate a 30-day CR exercise program in accordance with FITT-VP principles (Frequency, Intensity, Time, Type, Volume, Progression), standard safety considerations, and current American Heart Association/European Society of Cardiology (AHA/ESC) guideline recommendations. Two experienced CR specialists independently evaluated the generated programs for adherence to guidelines, safety, and clinical plausibility.

**Results:**

The model successfully generated unique 30-day exercise programs for all five profiles, incorporating warm-up and cool-down phases, aerobic and resistance training components, intensity guidance using rating of perceived exertion and/or heart rate targets, and progressive adaptation over time. The programs demonstrated appropriate condition-specific modifications, such as lower-intensity exercise for recent myocardial infarction and heart failure with reduced ejection fraction, and avoidance of high-intensity exercise bursts in atrial fibrillation. Expert evaluation confirmed broad adherence to the FITT-VP principles and the major safety recommendations of the current AHA/ESC guidelines. No overtly unsafe prescriptions were identified.

**Conclusion:**

DeepSeek was able to generate structured, guideline-informed exercise prescriptions across various simulated cardiac rehabilitation scenarios. These results suggest the model's capacity to generate guideline-informed exercise prescriptions aligned with current guideline principles in simulated settings, but they do not prove clinical effectiveness, safety, or suitability for routine practice. Additional studies involving real patients and direct comparisons with clinician-made exercise prescriptions are needed before clinical use can be considered.

## 1 Introduction

Cardiovascular diseases (CVD) remain the leading cause of morbidity and mortality worldwide, representing a major public health challenge (1). Cardiac rehabilitation (CR) is a cornerstone of secondary prevention and has been consistently shown to reduce hospital readmissions, improve functional capacity, enhance quality of life, and decrease cardiovascular mortality across a range of cardiac conditions (2–4). A central component of CR is structured exercise training tailored to the patient's clinical status, risk profile, and functional capacity.

International guidelines from the European Society of Cardiology (ESC) (5) and American College of Sports Medicine (6) provide comprehensive recommendations for exercise prescription, typically structured around the FITT-VP principles—frequency, intensity, time, type, volume, and progression (7, 8). Despite these frameworks, translating guideline recommendations into individualized and scalable programs remains challenging in routine practice due to time constraints, variability in clinician expertise, and increasing demand for both center-based and home-based rehabilitation (9).

Digital health tools are increasingly being explored to support rehabilitation care pathways. Among these, artificial intelligence (AI), particularly large language models (LLMs), has recently emerged as a potential tool for structured clinical support tasks. These systems are capable of generating contextually relevant outputs based on large-scale training data and have shown promise in healthcare applications, including documentation, decision support, and patient education (10, 11).

In the context of cardiac rehabilitation, AI tools can support the development of standardized, guideline-based exercise plans that clinicians may subsequently review and adapt for individual patients (12–15). This is particularly relevant given persistent challenges with participation and adherence to CR worldwide, underscoring the need for more efficient, scalable rehabilitation delivery models.

Despite this potential, evidence regarding the use of LLMs specifically for exercise prescription in cardiac rehabilitation remains limited. In particular, it is unclear whether AI-generated programs can adequately reflect condition-specific precautions, appropriate progression, and key safety principles across different cardiac populations commonly encountered in clinical practice. Furthermore, evidence comparing AI-generated exercise prescriptions with clinician-designed programs remains lacking.

This study aimed to assess the characteristics of AI-generated exercise prescriptions, including adherence to guidelines, expert-evaluated safety, and clinical plausibility, across five representative cardiac rehabilitation scenarios. We hypothesized that the model would generate structured, safe, and guideline-concordant baseline programs.

## 2 Materials and methods

### 2.1 Study design

This was a simulation-based evaluation of AI-generated exercise prescriptions across five representative cardiac rehabilitation scenarios. The study was conducted between January and March 2026 and followed a two-phase design. First, the study team developed five standardized clinical profiles representing common cardiac rehabilitation referrals. Second, the DeepSeek-V3 large language model was prompted to generate a 30-day exercise prescription for each profile using a standardized prompt based on FITT-VP principles and current guideline recommendations. The generated prescriptions were independently evaluated by two experienced cardiac rehabilitation specialists for adherence to guidelines, expert-assessed safety, and clinical plausibility. Each domain was rated using a 5-point Likert scale. The final quantitative score for each domain was calculated as the mean of the two reviewers' ratings. Following the independent scoring, the reviewers discussed any substantial discrepancies to reach a shared qualitative interpretation of the strengths and limitations of each exercise prescription. The study was conducted entirely in English.

### 2.2 Clinical profiles

Five distinct, purpose-designed clinical profiles were developed by the study team to represent common referral scenarios in CR setting:

1. **Recent Myocardial Infarction (MI) (<1 month):** 55-year-old male, sedentary lifestyle, 2 weeks post-anterior ST-segment Elevation Myocardial Infarction (STEMI) treated with percutaneous coronary intervention (PCI) to the left anterior descending artery (LAD), left ventricular ejection fraction (LVEF) 50%, stable hemodynamics, no residual ischemia, and no significant arrhythmia.
2. **Prior MI (>1 month):** 60-year-old female, 6 months post—Non-ST-segment Elevation Myocardial Infarction (NSTEMI) (medically managed), LVEF 55%, previously active but reluctant to exercise post-MI, and no angina.
3. **Atrial fibrillation (AF):** 65-year-old male, paroxysmal AF, rate-controlled on beta-blocker therapy, CHA₂DS_2_-VASc score 3, no significant structural heart disease, LVEF 60%.
4. **Heart Failure with Reduced Ejection Fraction** (**HFrEF):** 70-year-old female, ischemic cardiomyopathy, LVEF 35%, New York Heart Association (NYHA) Class II symptoms, stable on guideline-directed medical therapy.
5. **Heart Failure with Preserved Ejection Fraction** (**HFpEF):** 68-year-old male, history of hypertension and diabetes, LVEF 50%, evidence of diastolic dysfunction, NYHA Class II symptoms (predominantly dyspnea on exertion) (Table 1).

These profiles were intentionally constructed to capture clinical heterogeneity in diagnosis, symptom burden, and exercise-related precautions while remaining representative of common cardiac rehabilitation referrals. However, they do not encompass the full spectrum of clinical complexity encountered in routine practice.

**Table 1:** Standardized Clinical Scenarios.

Condition	Age	Clinical Characteristics
Recent MI	55	2 weeks post STEMI, clinically stable
Prior MI	60	6 months post NSTEMI clinically stable
Atrial fibrillation	65	Paroxysmal AF
HFrEF	70	EF 35% NYHA II
HFpEF	68	EF 55% hypertension

Clinical characteristics of standardized cardiovascular scenarios used in the AI exercise prescription evaluation.

### 2.3 AI model and prompting

All interactions were conducted through the public web interface of DeepSeek (DeepSeek Inc., Hangzhou, China, (https://chat.deepseek.com). The model employed was DeepSeek-V3, an LLM available at the time of the study. Sessions were performed in March 2026 using the default settings provided through the web interface. The model's reported knowledge cutoff was May 2025.

A standardized prompt template was applied across all profiles to ensure methodological consistency and reproducibility. The prompts incorporated FITT-VP exercise prescription principles, safety considerations, and recommendations. The full prompt template and exercise programs for each scenario are provided in Supplementary Material 1. For each clinical scenario, the model received information regarding the patient's diagnosis, age, and relevant clinical characteristics (e.g., ejection fraction, time since cardiac event, and NYHA functional class, where applicable). The following core instruction: “Design a detailed 30-day cardiac rehabilitation exercise program for this patient. The program must adhere strictly to FITT-VP principles (5). Prioritize safety above all else. Include specific recommendations for warm-up (5–10 min), aerobic exercise (type, duration, intensity using RPE Borg 6–20 scale, and/or % HRmax target, frequency), resistance training (type, sets, reps, frequency, ensuring safety), cool-down (5–10 min), and flexibility. Specify gradual progression over the 30 days. Incorporate condition-specific modifications and precautions based on current AHA/ESC guidelines. Provide the Rate of Perceived Exertion (RPE) targets clearly”.

The prompt explicitly required a gradual progression of exercise parameters over the 30-day period, in line with standard cardiac rehabilitation principles.

**Figure 1:**
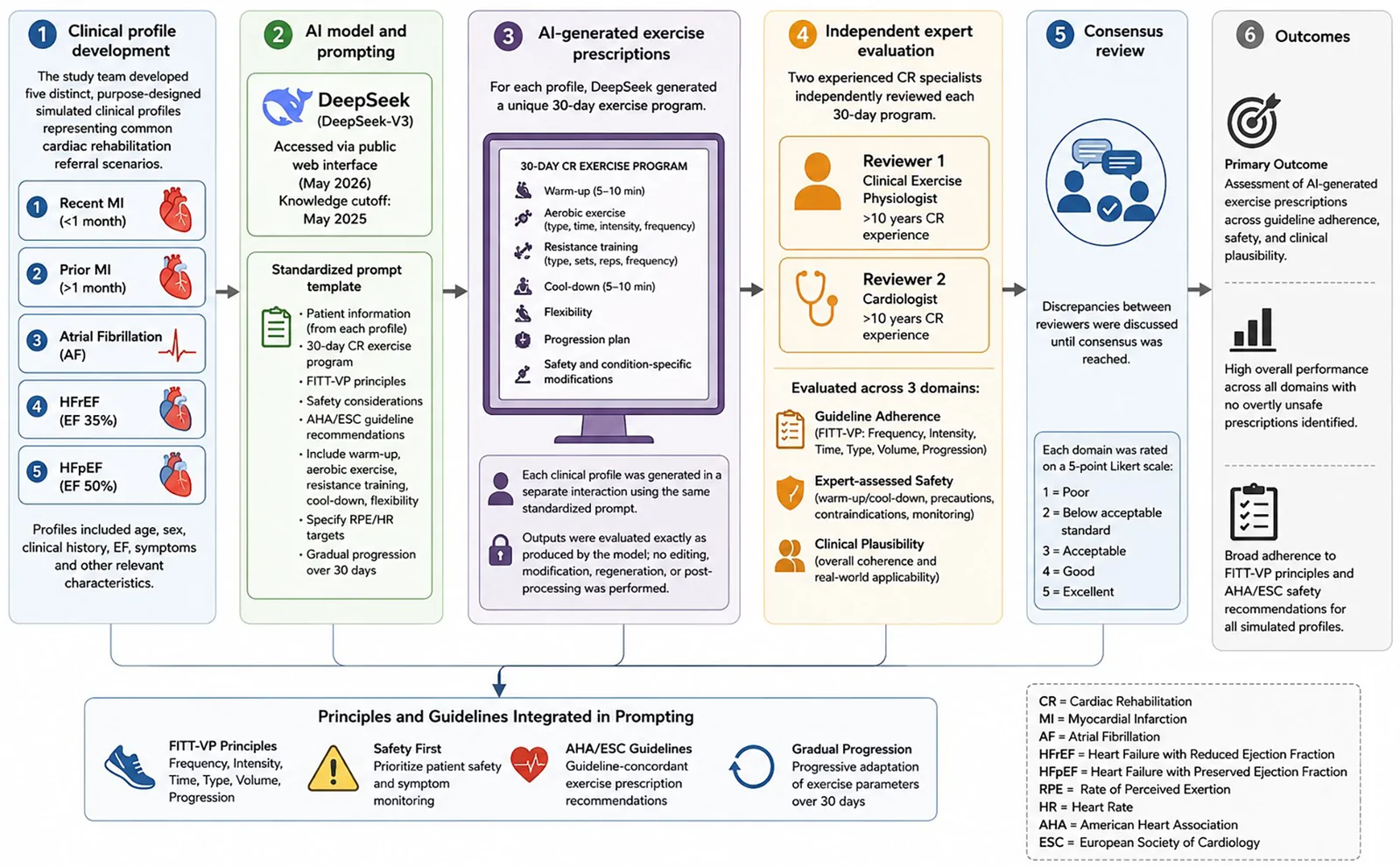
Study workflow. Overview of the study design and evaluation process for Al-generated exercise prescriptions in five simulated cardiac rehabilitation clinical scenarios.

Each clinical profile was generated in a separate interaction, using the same standardized prompt, and conducted by a single investigator to ensure consistency. AI-generated outputs were evaluated exactly as produced by the model; no editing, modification, regeneration, or post-processing was performed before expert assessment. Figure 1 illustrates the overall study workflow, from the development of the five representative clinical scenarios and standardized prompting procedure to the generation of AI-based exercise prescriptions and their subsequent independent expert evaluation. The figure summarizes the study's sequential phases and the assessment process used across all simulated scenarios.

### 2.4 Evaluation procedure and outcomes

Two experienced CR specialists independently reviewed each model-generated 30-day program. Reviewer 1 was a clinical exercise physiologist, and Reviewer 2 was a cardiologist; both had more than 10 years of experience in CR. Programs were evaluated across three domains:

- **Guideline Adherence:** Appropriateness of prescribed Frequency, Intensity (RPE/HR targets relative to condition, Time, Type (aerobic, resistance, flexibility), Volume, and Progression.
- **Expert-assessed Safety:** Inclusion of appropriate warm-up/cool-down, identification of contraindicated activities (e.g., high-intensity bursts for AF, Valsalva for recent MI), inclusion of necessary precautions (e.g., hydration, symptom monitoring, weight monitoring for HF).
- **Clinical Plausibility:** Overall coherence of the program in a real-world CR setting. Discrepancies between reviewers were discussed until consensus was reached.

Each domain was rated using a 5-point Likert scale (1 = poor, 2 = below acceptable standard, 3 = acceptable, 4 = good, 5 = excellent). For each domain, the final quantitative score was calculated as the mean of the two cardiac rehabilitation specialists' ratings.

### 2.5 Outcomes

The primary outcome was the assessment of AI-generated exercise prescriptions across the three predefined domains: guideline adherence, safety, and clinical plausibility.

### 2.6 Ethics

Ethical approval was not required because the study involved only hypothetical, non-identifiable clinical profiles and did not include human participants or patient data.

## 3 Results

The model successfully generated distinct, structured 30-day exercise programs for all five clinical profiles. Each program included all core components of cardiac rehabilitation exercise prescription, including warm-up and cool-down phases, aerobic training, resistance exercise where appropriate, flexibility components, explicit intensity guidance based on rating of perceived exertion (RPE) and/or heart rate targets, and progressive adaptation over time. The study workflow is summarized in Figure 1.

### 3.1 Guideline adherence, expert-assessed safety, and clinical plausibility

Quantitative expert evaluation demonstrated high overall performance across all domains. Mean scores were 4.5 for guideline adherence, 5.0 for safety, and 4.3 for clinical plausibility.

Across all five profiles, expert review confirmed broad adherence to FITT-VP principles and the absence of manifestly unsafe recommendations within the context of the simulated clinical scenarios. Programs were generally conservative in their initial intensity, appropriately structured in their progression, and considered clinically plausible by the expert reviewers.

Safety received the highest expert rating across all domains, with consistently high ratings across all profiles (range: 4.8–5.0), and four out of five scenarios achieved the maximum score. Guideline adherence was also rated highly (range: 4.3–4.7), indicating appropriate implementation of exercise prescription principles. Clinical plausibility, while still rated positively, showed slightly greater variability (range: 4.1–4.4), reflecting differences in the level of contextual personalization across clinical scenarios (Table 2).

**Table 2:** Expert Evaluation Scores.

Condition	Guideline adherence	Safety	Clinical plausibility
Recent MI	4.5	5.0	4.2
Prior MI	4.7	5.0	4.4
Atrial fibrillation	4.3	4.8	4.1
HFrEF	4.4	5.0	4.3
HFpEF	4.6	5.0	4.4

Mean expert evaluation scores for AI generated exercise prescriptions across clinical scenarios.

Consensus review indicated that safety performance was driven by consistent inclusion of warm-up and cool-down phases, appropriate symptom-monitoring instructions, and avoidance of contraindicated activities. Guideline adherence was generally strong, although minor refinements were occasionally required in relation to progression structure and personalization. Clinical plausibility was considered acceptable to good across all profiles, with the recognition that the generated programs represent baseline templates rather than fully individualized prescriptions.

### 3.2 Condition-Specific findings

#### 3.2.1 Recent MI (<1 month)

The program correctly emphasized a very conservative start: Frequency 3–5 days/week (initially mostly aerobic); Intensity RPE 9 10–15 min progressing to 20–30 min; Type low-impact aerobics (walking, stationary cycling), light resistance introduced later (e.g., bands, 1–2 lbs weights, 1 set × 10–15 reps).

Analysis*:* AI correctly prioritized low intensity and gradual progression, crucial for myocardial healing and preventing strain.

#### 3.2.2 Prior MI (>1 month)

The program allowed for moderate intensity: Frequency 5 days/week (3 aerobic, 2 resistance); Intensity RPE 12–14/20 (moderate); Time 20–40 min aerobic; Type brisk walking, cycling, swimming; Resistance training included (e.g., light-moderate weights, 2 sets × 10–15 reps). Analysis*:* Appropriate progression reflecting a more stable phase post-MI, aiming to improve cardiorespiratory fitness.

#### 3.2.3 Atrial fibrillation

The program focused on regular, moderate-intensity aerobic exercise: Frequency 5 days/week; Intensity RPE 11–13/20; Time 30 min; Type continuous aerobic (walking, elliptical, cycling), avoiding sudden high-intensity bursts. Flexibility and balance exercises were included.

Analysis*:* AI correctly avoided high-intensity intervals and emphasized consistency, aligning with recommendations to minimize arrhythmia triggers.

#### 3.2.4 HFrEF (EF 35%)

The program prescribed lower to moderate intensity with optional intervals: Frequency 4–5 days/week; Intensity RPE 11–13/20; Time initially shorter bouts (e.g., 10–15 min) progressing to 30 min, potentially using interval training (e.g., 5 min work/2 min rest); Type walking, recumbent cycling; Light resistance included. Crucially, it included advice on daily weight monitoring.

Analysis*:* The focus on moderate intensity, interval options, and weight monitoring reflects key management strategies for HFrEF.

#### 3.2.5 HFpEF (EF 50%)

The program was similar to HFrEF but allowed slightly higher intensity ranges within the moderate spectrum: Frequency 5 days/week; Intensity RPE 12–14/20; Time 30–40 min; Type aerobic (walking, swimming, cycling), resistance training. Emphasis on adequate warm-up/cool-down and managing dyspnea. Analysis*:* Program targeted improving exercise tolerance and managing symptoms, appropriate for HFpEF pathophysiology.

## 4 Discussion

This simulation study evaluated the characteristics of AI-generated exercise prescriptions, including adherence to guidelines, expert-evaluated safety, and clinical plausibility across multiple clinical scenarios. The findings indicate that this large language model was able to generate structured 30-day exercise programs that were rated by experts as free of manifestly unsafe recommendations within the context of the simulated clinical profiles and broadly aligned with established exercise prescription principles.

One of the most relevant findings of this study is the consistently high expert-rated safety observed across all clinical scenarios. The model reliably incorporated key safety elements, including warm-up and cool-down phases, conservative initial intensity, gradual progression, and clear symptom monitoring recommendations. This is particularly important in CR, where inappropriate exercise prescription may increase the risk of adverse events. The ability of the model to consistently reproduce these safety components suggests that core safety principles could be effectively translated into structured outputs, even in the absence of real-time clinical data.

In parallel, expert review indicated strong adherence to guideline-based exercise prescription frameworks. The consistent implementation of FITT-VP principles across heterogeneous cardiovascular conditions indicates that LLMs can synthesize and operationalize structured clinical knowledge. This observation is consistent with emerging evidence showing that advanced LLMs can generate clinically relevant outputs in structured medical tasks. For instance, Lee et al. reported that GPT-4 can produce clinically coherent responses in complex medical scenarios, although requiring expert validation (16). Similarly, Thirunavukarasu et al. highlighted the potential of LLMs to support clinical workflows while emphasizing limitations in nuanced clinical reasoning (17).

These findings are further supported by recent evaluations of LLMs in medical assessment tasks, which demonstrate strong performance in structured, knowledge-based scenarios but reduced accuracy in contexts requiring deeper contextual adaptation (13).

Despite these strengths, clinical plausibility scores were slightly lower, and more variable compared to safety and guideline adherence. This likely reflects the intrinsic limitation of current generative AI systems in capturing the full complexity of individualized clinical decision-making. Exercise prescription in cardiac rehabilitation requires continuous adaptation based on patient-specific factors, including comorbidities, functional capacity, psychosocial context, adherence, and real-time physiological responses. The use of static, prompt-based inputs limits the model's ability to account for these dynamic and context-dependent variables, resulting in outputs that are appropriate as baseline templates but not fully individualized rehabilitation plans. This limitation is consistent with prior work emphasizing that AI systems, while effective in processing structured information, remain less capable of integrating complex, patient-centered variables into decision-making processes (14).

These findings align with the broader perspective in the literature that positions artificial intelligence as a tool to augment, rather than replace, clinical expertise. As highlighted by Topol, the future of healthcare is likely to be shaped by the integration of human clinical judgment with AI-supported systems (11). Within this framework, AI-generated exercise prescriptions may serve as an initial structured foundation, allowing clinicians to focus on higher-level tasks such as personalization, patient engagement, and adaptive clinical decision-making.

From a clinical implementation standpoint, these results have relevant implications for cardiac rehabilitation delivery. Participation in CR programs remains suboptimal worldwide, with well-documented barriers including limited access, resource constraints, and variability in service availability. Digital health interventions have been proposed to improve access to and adherence with rehabilitation programs (15, 18, 19). The present findings extend this body of evidence by suggesting that AI-generated exercise prescriptions may represent an additional layer of support within digitally enabled rehabilitation pathways, particularly in hybrid and home-based care models.

However, several limitations must be acknowledged. This study was based on hypothetical clinical scenarios, which do not fully capture the complexity of real-world patient management. Furthermore, only five representative simulated clinical scenarios were evaluated. Although these profiles reflect common cardiac rehabilitation indications, they do not cover the full range of patients seen in routine clinical practice. Therefore, the findings should not be considered applicable to all cardiac rehabilitation populations, and future studies should include more diverse and clinically complex patient profiles. The generated prescriptions were evaluated under simulated conditions and were not tested in actual patients. Consequently, no conclusions can be drawn regarding clinical effectiveness, patient adherence, or long-term outcomes. In addition, the AI operated exclusively on static prompt-based information and was unable to incorporate real-time physiological data, longitudinal responses to training, or dynamic clinical changes. Exercise prescription in cardiac rehabilitation requires continuous adaptation according to symptoms, functional capacity, treatment response, comorbidities, and patient preferences, factors that current large language models cannot fully integrate.

Another limitation relates to the evaluation methodology, which relied on expert judgment using Likert-scale ratings. Although independent assessments and subsequent consensus discussions were performed to improve consistency, this approach remains partially subjective. Future studies should incorporate more objective and standardized assessment frameworks, as well as clinically relevant outcome measures. A further important limitation is the lack of a direct comparison with clinician-generated exercise prescriptions. As a result, this study cannot determine whether AI-generated recommendations are comparable to those developed by experienced cardiac rehabilitation professionals or evaluate the added benefit of AI-assisted exercise prescription. Future studies should include direct comparisons with clinician-designed programs. Finally, only a single large language model was evaluated, limiting the generalizability of the findings to other AI systems. Although no overtly unsafe recommendations were identified in the simulated scenarios examined, large language models remain susceptible to hallucinations, namely the generation of inaccurate, fabricated, or unsupported information presented with apparent confidence. In the context of cardiac rehabilitation, such outputs could potentially result in inappropriate exercise recommendations if accepted without critical clinical review. Therefore, clinician oversight remains essential for any potential application of these tools in clinical practice.

These limitations highlight important directions for future research. Prospective clinical studies are needed to evaluate the impact of AI-assisted exercise prescription on patient outcomes. Integration with wearable technologies and remote monitoring systems may enable dynamic adjustment of exercise programs based on physiological responses, improving personalization, and safety. Furthermore, the development of more transparent and interpretable AI systems will be essential to enhance clinician trust and facilitate safe integration into clinical workflows. To our knowledge, this is among the first studies specifically evaluating the application and safety of the LLM DeepSeek for exercise prescription within cardiac rehabilitation, addressing an important gap in the current literature.

## 5 Conclusion

This study suggests that DeepSeek was able to generate structured exercise prescriptions for a range of cardiac rehabilitation scenarios. Expert reviewers judged these prescriptions to be broadly consistent with guideline-based exercise prescription principles and free of overtly unsafe recommendations within the simulated cases.

However, these findings should be interpreted as an assessment of the model's ability to generate structured, guideline-informed exercise prescriptions under simulated conditions rather than evidence of clinical effectiveness, safety, or applicability in routine practice. Further research is needed to compare AI-generated and clinician-generated exercise prescriptions and to evaluate the clinical effectiveness, safety, and real-world applicability of these approaches in prospective patient studies.

## Data Availability

The original contributions presented in the study are included in the article/Supplementary Material, further inquiries can be directed to the corresponding author.
