# Supplementary material for: Evaluation of AI-generated exercise prescriptions for diverse cardiac conditions in rehabilitation: a simulation study using the DeepSeek model
Source: Front Rehabil Sci. 2026 Aug 18;7:1844420. doi: 10.3389/fresc.2026.1844420 (PMC13529687; doi:10.3389/fresc.2026.1844420)
Supplement: Supplementary file 1 [file Datasheet1.docx]

**SUPPLEMENTARY MATERIAL 1**

**PROMPT**

Design a detailed 30-day cardiac rehabilitation exercise program for these patient profiles. The program must adhere strictly to FITT-VP principles. Prioritize safety above all else. Include specific recommendations for warm-up (5-10 min), aerobic exercise (type, duration, intensity using RPE Borg 6-20 scale, and/or %HRmax target, frequency), resistance training (type, sets, reps, frequency, ensuring safety), cool-down (5-10 min), and flexibility. Specify a gradual progression over the 30 days. Incorporate condition-specific modifications and precautions based on current AHA/ESC guidelines. Provide the Rate of Perceived Exertion (RPE) targets clearly:

**Patient 1**

**Recent MI (<1 month):** 55-year-old male, sedentary lifestyle, 2 weeks post-anterior ST-segment Elevation Myocardial Infarction (STEMI) treated with percutaneous coronary intervention (PCI) to the left anterior descending artery (LAD), left ventricular ejection fraction (LVEF) 50%, stable hemodynamics, no residual ischemia, and no significant arrhythmia

\# 30-Day Cardiac Rehabilitation Exercise Program

\## Post-STEMI Patient with PCI to LAD

---

\### Patient Profile Summary

| Characteristic | Detail |

|----------------|--------|

| Age/Sex | 55-year-old male |

| Condition | Anterior STEMI, 2 weeks post-PCI to LAD |

| LVEF | 50% |

| Risk Level | Moderate (LVEF 40-49% would be higher risk; 50% with stable hemodynamics is favorable) |

| Activity Status | Sedentary lifestyle prior to event |

| Key Precautions | Recent stent placement, deconditioning, post-MI scar maturation |

---

\## Program Design: FITT-VP Framework

The FITT-VP principle (Frequency, Intensity, Time, Type, Volume, Progression) forms the foundation of this program, as recommended by the American College of Sports Medicine (ACSM) and major cardiovascular societies .

---

\## Weeks 1-2: Foundation Phase (Days 1-14)

\### Focus: Establish baseline tolerance, monitor response, build confidence

---

\#### Warm-Up (5-10 minutes each session)

- **Type**: Low-intensity rhythmic movements

- **Activities**:

- Seated or standing marching in place (1-2 min)

- Arm circles (small to large, forward/backward)

- Gentle trunk rotations

- Ankle pumps and toe raises

- **Intensity**: RPE 6-8 (very light)

- **Purpose**: Gradual increase in heart rate and blood flow; prepares cardiovascular system for exercise load

---

\#### Aerobic Exercise

| Component | Specification |

|-----------|---------------|

| **Type** | Treadmill walking OR Stationary cycling (recumbent preferred initially) |

| **Frequency** | 5 days/week |

| **Duration** | Start: 10-15 minutes continuous |

| **Intensity** | RPE 10-12 (light to somewhat hard) |

| **%HRmax Target** | 40-55% HRmax (~72-80 bpm if HRmax ~180) |

| **Monitoring** | Heart rate, rhythm, BP before and after, symptoms |

**Weeks 1-2 Progression:**

- Days 1-3: 10 minutes at RPE 10

- Days 4-7: 12 minutes at RPE 10-11

- Days 8-10: 15 minutes at RPE 11

- Days 11-14: 15-18 minutes at RPE 11-12

> **⚠️ Safety Cue**: Stop immediately for chest pain, dizziness, unusual shortness of breath, palpitations, or irregular heartbeat

---

\#### Resistance Training

| Component | Specification |

|-----------|---------------|

| **Type** | Bodyweight and light resistance band exercises |

| **Frequency** | 2 non-consecutive days/week (e.g., Monday & Thursday) |

| **Sets/Reps** | 1 set × 10-12 repetitions per exercise |

| **Intensity** | RPE 10-11 (light) |

| **Rest** | 2 minutes between exercises |

**Exercises (Weeks 1-2):**

| Exercise | Purpose | Notes |

|----------|---------|-------|

| Wall sit (30-45 sec) | Lower body strength | Keep back flat against wall |

| Seated leg extensions (no weight) | Quadriceps | Full extension, controlled |

| Standing heel raises (holding chair) | Calf strength | Slow tempo |

| Seated bicep curls (1-2 lbs or light band) | Upper body | **No straining; breathe out on lift** |

| Shoulder external rotation (band) | Rotator cuff | Elbows at 90°, gentle |

> **⚠️ Resistance Training Precautions**:

> - **Do not begin resistance training until 3-4 weeks post-MI**

> - **Breathe out during exertion, inhale on return** — avoid Valsalva maneuver

> - **Keep elbows slightly bent** when lifting

> - **Count 2 seconds lifting, 4 seconds lowering**

---

\#### Cool-Down & Flexibility (5-10 minutes each session)

| Component | Specification |

|-----------|---------------|

| **Type** | Static stretching; gradual reduction in intensity |

| **Activities** | Seated hamstring stretch, chest stretch (gentle), calf stretch, shoulder rolls |

| **Intensity** | RPE 6-8; hold stretches 15-30 seconds |

| **Purpose** | Prevent blood pooling, reduce muscle tension, facilitate recovery |

---

\## Weeks 3-4: Progression Phase (Days 15-30)

\### Focus: Increase exercise capacity, introduce more challenging resistance

---

\#### Aerobic Exercise

| Component | Specification |

|-----------|---------------|

| **Type** | Treadmill walking, stationary cycling, OR elliptical trainer |

| **Frequency** | 5-7 days/week |

| **Duration** | 20-30 minutes continuous |

| **Intensity** | RPE 12-14 (somewhat hard) |

| **%HRmax Target** | 55-70% HRmax (~99-126 bpm if HRmax ~180) |

**Weeks 3-4 Progression:**

- Days 15-18: 18-20 minutes at RPE 11-12

- Days 19-22: 20-22 minutes at RPE 12-13

- Days 23-26: 22-25 minutes at RPE 13

- Days 27-30: 25-30 minutes at RPE 13-14

> **Note**: Some patients may tolerate 30 minutes at RPE 12-13 by the end of Week 4; adjust based on daily assessment

---

\#### Resistance Training

| Component | Specification |

|-----------|---------------|

| **Type** | Light dumbbells (1-3 lbs arms, 3-5 lbs legs) + bands |

| **Frequency** | 2-3 non-consecutive days/week |

| **Sets/Reps** | 1-2 sets × 12-15 repetitions per exercise |

| **Intensity** | RPE 11-13 (light to somewhat hard) |

| **Rest** | 2-3 minutes between sets |

**Exercises (Weeks 3-4):**

| Exercise | Sets/Reps | Notes |

|----------|-----------|-------|

| Wall squat (45-60 sec) | 1-2 × 12-15 | Progress from wall sit |

| Leg press (light) OR seated leg extensions | 1-2 × 12-15 | If machine available |

| Standing calf raises (holding support) | 1-2 × 12-15 | Use light hand weights if stable |

| Bicep curls (1-3 lbs) | 1-2 × 12-15 | Slow, controlled |

| Overhead press (1-2 lbs) — **optional** | 1 × 12 | **Monitor BP; avoid if dizziness** |

| Rows (band or light weight) | 1-2 × 12-15 | Posture focus |

| Core: Modified plank (wall) OR seated trunk flexion | 1 × 10-12 | Protect lower back |

> **⚠️ Upper Body Caution**: Avoid vigorous upper body exercise or lifting heavy weights for 4-6 weeks post-stent placement; chest wall and sternal precautions apply

---

\## Daily Session Structure (Applies to All Days)

```

┌─────────────────────────────────────────────────────────────┐

│	SESSION STRUCTURE	│

├─────────────────────────────────────────────────────────────┤

│ 1. WARM-UP (5-10 min)	│

│	├── Light marching/cycling (RPE 6-8)	│

│	└── Dynamic stretching	│

│	│

│ 2. AEROBIC PHASE (10-30 min)	│

│	├── Target: RPE 10-14 per phase	│

│	├── Monitor: HR, symptoms, rhythm	│

│	└── Stop if warning signs appear	│

│	│

│ 3. RESISTANCE TRAINING (10-15 min)	│

│	├── 2-3 days/week (non-consecutive)	│

│	├── 1-2 sets × 12-15 reps (Weeks 3-4)	│

│	└── RPE 10-13	│

│	│

│ 4. COOL-DOWN (5-10 min)	│

│	├── Gradual intensity reduction	│

│	└── Static stretching (15-30 sec holds)	│

└─────────────────────────────────────────────────────────────┘

```

---

\## RPE Target Summary (Borg 6-20 Scale)

| Phase | Warm-Up | Aerobic | Resistance | Cool-Down |

|-------|---------|---------|------------|-----------|

| Weeks 1-2 | 6-8 | 10-12 | 10-11 | 6-8 |

| Weeks 3-4 | 6-9 | 12-14 | 11-13 | 6-9 |

**Borg RPE Scale Reference:**

- 6 = No exertion at all

- 7-8 = Extremely light

- 9 = Very light

- 10-11 = Light

- 12-13 = Somewhat hard

- 14-15 = Hard

- 16-17 = Very hard

- 18-19 = Extremely hard

- 20 = Maximal exertion

---

\## Condition-Specific Modifications & Precautions

\### Based on AHA/ESC Guidelines for Post-MI Cardiac Rehabilitation

1. **Exercise is recommended Class IA for STEMI patients** — exercise-based cardiac rehabilitation is indicated for all patients with ST-segment elevation MI

2. **Monitoring requirements** :

- **Heart rate, BP, rhythm, and symptoms** before, during, and after each session

- **Continuous ECG monitoring** during supervised sessions (first 2-4 weeks)

- **Oxygen saturation** monitoring as indicated

3. **LAD PCI specific considerations**:

- Anterior MI with LVEF 50% → moderate risk; monitor for **heart failure symptoms** (dyspnea, edema, fatigue)

- **No vigorous upper body exercise** for 4-6 weeks post-stent placement

- Avoid **isometric or heavy lifting** that may cause Valsalva

4. **Warning signs requiring immediate cessation**:

- Chest pain/discomfort

- Dizziness or lightheadedness

- Unexpected shortness of breath

- Palpitations or irregular heartbeat

- Excessive fatigue

- Clicking sensation in chest (sternal precautions)

5. **Medication considerations**:

- Keep **nitroglycerin** (if prescribed) accessible during exercise

- Ensure BP is stable before exercise — avoid if SBP < 90 or > 180 mmHg

- Beta-blocker therapy may blunt HR response — **rely primarily on RPE** for intensity guidance

---

\## Weekly Summary Schedule

| Day | Aerobic | Resistance | Notes |

|-----|---------|------------|-------|

| **Monday** | ✓ (20-30 min) | ✓ (Weeks 3-4) | Supervised if possible |

| **Tuesday** | ✓ | — | |

| **Wednesday** | ✓ | ✓ (Weeks 3-4) | |

| **Thursday** | ✓ | — | |

| **Friday** | ✓ | ✓ (Weeks 3-4) | |

| **Saturday** | ✓ | — | Optional: light walking |

| **Sunday** | Rest or light walking | — | Active recovery |

---

\## Key References

- AHA/ACC Guideline: STEMI patients should undergo exercise-based cardiac rehabilitation (Class IA)

- ACSM FITT-VP principle for exercise prescription

- Resistance training may begin 3-4 weeks post-MI with light resistance (1-3 lbs)

- Moderate aerobic intensity: RPE 12-13 on Borg 6-20 scale

---

*This program is designed for medical supervision. All patients should undergo formal cardiopulmonary exercise testing (CPET) prior to initiating unsupervised exercise and should be cleared by their cardiologist. Adjustments should be made based on individual response, symptoms, and daily clinical assessment.*

**Patient 2**

Prior MI (>1 month): 60-year-old female, 6 months post – Non-ST-segment Elevation Myocardial Infarction (NSTEMI) (medically managed), LVEF 55%, previously active but reluctant to exercise post-MI, and no angina

\# 30-Day Cardiac Rehabilitation Exercise Program

\## Patient Profile Summary

- **Demographic:** 60-year-old female

- **Diagnosis:** Non-ST-segment Elevation Myocardial Infarction (NSTEMI), 6 months post-event, medically managed

- **Cardiac Function:** LVEF 55% (preserved ejection fraction)

- **Risk Profile:** No angina, previously active but now reluctant to exercise post-MI

- **Key Considerations:** Psychological barrier (fear/reluctance), deconditioning risk, excellent cardiac function reserve

---

\## Program Overview

This program follows **FITT-VP principles** (Frequency, Intensity, Time, Type, Volume, Progression) and prioritizes safety through gradual progression, symptom monitoring, and appropriate RPE targets.

\### Fundamental Safety Precautions (AHA/ESC Guidelines)

**STOP EXERCISE AND SEEK MEDICAL ATTENTION if experiencing:**

- Chest pain, pressure, tightness, or burning

- Unusual aching in arms, shoulders, neck, jaw, or back

- Dizziness, lightheadedness, or nausea

- Racing or skipping heartbeat

- Unusual shortness of breath or difficulty breathing

- Extreme fatigue

**Pre-Exercise Checklist:**

- Confirm resting heart rate within target range (60-100 bpm)

- Confirm resting systolic BP between 90-160 mmHg

- No new or worsening symptoms in the past 24 hours

- Take prescribed medications as directed

---

\## Week 1: Foundation and Reassurance (Days 1-7)

\### Key Focus

Establish routine, rebuild confidence, assess baseline tolerance, overcome exercise reluctance through gentle initiation.

\### Warm-Up (5-10 minutes)

| Activity | Duration | Details |

|----------|----------|---------|

| Low-intensity walking | 3-5 min | Gentle pace in place or around room |

| Arm circles | 1-2 min | Small circles progressing to larger |

| Shoulder rolls | 1 min | Forward and backward |

| Neck stretches | 1 min | Gentle side-to-side |

| Ankle pumps | 1 min | Point and flex |

\### Aerobic Exercise

| Component | Specification |

|-----------|---------------|

| **Frequency** | 3 days/week (e.g., Monday, Wednesday, Friday) |

| **Type** | Walking (indoor or flat outdoor surface) or stationary cycling |

| **Duration** | 15-20 minutes continuous or 2 x 10-minute bouts |

| **Intensity** | RPE 9-11 (Very Light to Light); 40-50% HRmax (~96-105 bpm) |

| **Monitoring** | Talk test: able to speak in full sentences |

**RPE Target:** 9-11 (Borg 6-20 scale)

- "Very Light" to "Light" exertion

- Patient should feel she is working, but conversation remains comfortable

\### Resistance Training (Circuit Format)

| Exercise | Sets | Reps | Notes |

|----------|------|------|-------|

| Wall push-ups | 1 | 8-10 | Hands shoulder-width apart, feet 2 feet from wall |

| Seated leg raises | 1 | 10-12 | Alternating legs, slow and controlled |

| Seated bicep curls | 1 | 8-10 | Use 1-2 lb weights or resistance bands |

| Seated rows | 1 | 8-10 | Use resistance band anchored at chest height |

| Seated marches | 1 | 10-12 | Alternate legs, keep core engaged |

**Resistance Training Guidelines:**

- No breath-holding (exhale on exertion)

- Maintain 4-second repetition cadence (1 second concentric, 3 seconds eccentric)

- **Frequency:** 2 days/week (Tuesday, Thursday)

\### Cool-Down (5-10 minutes)

| Activity | Duration | Details |

|----------|----------|---------|

| Gentle walking | 2-3 min | Gradual slowing of pace |

| Standing quad stretch | 1 min each | Hold onto chair for balance |

| Standing calf stretch | 1 min each | Against wall |

| Seated trunk rotation | 1 min | Gentle side-to-side |

| Deep breathing | 2-3 min | 5-second inhale, 5-second exhale |

\### Flexibility

- Perform during cool-down, holding each stretch for 30 seconds

- Focus: hamstrings, quadriceps, chest, shoulders

- No bouncing; feel mild tension, not pain

---

\## Week 2: Building Consistency (Days 8-14)

\### Key Focus

Increase exercise duration, maintain moderate intensity, build exercise confidence.

\### Warm-Up (5-10 minutes)

Same as Week 1; may slightly increase pace during walking portion.

\### Aerobic Exercise

| Component | Specification |

|-----------|---------------|

| **Frequency** | 3-4 days/week |

| **Type** | Walking or stationary cycling; introduce alternate (e.g., swimming if available) |

| **Duration** | 20-25 minutes |

| **Intensity** | RPE 11-12 (Light to Somewhat Hard); 45-55% HRmax (~99-116 bpm) |

| **Monitoring** | Continue using talk test |

**RPE Target:** 11-12

- "Light" to low-end "Somewhat Hard"

- Breathing deeper but conversation still possible

\### Resistance Training

| Exercise | Sets | Reps | Progression |

|----------|------|------|-------------|

| Wall push-ups | 1-2 | 10-12 | Increase reps |

| Seated leg raises | 2 | 10-12 | Same |

| Seated bicep curls | 1-2 | 10-12 | Increase to 2-3 lb weights |

| Seated rows | 1-2 | 10-12 | Increase band resistance |

| Seated marches | 2 | 10-12 | Same |

| **NEW:** Hip abduction | 1 | 10-12 each | Seated, resistance band around thighs |

**Frequency:** 2 days/week (Tuesday, Thursday)

\### Cool-Down (5-10 minutes)

Same as Week 1; add chest stretch (gentle stretch for pectoral muscles).

\### Flexibility

Same as Week 1; hold each stretch 30-45 seconds.

---

\## Week 3: Progressive Overload (Days 15-21)

\### Key Focus

Increase session volume and introduce higher intensity intervals.

\### Warm-Up (5-10 minutes)

Same structure; may include gentle dynamic stretching.

\### Aerobic Exercise

| Component | Specification |

|-----------|---------------|

| **Frequency** | 4 days/week |

| **Type** | Primary: walking or cycling; Alternate: swimming, elliptical |

| **Duration** | 25-30 minutes |

| **Intensity** | RPE 12-13 (Somewhat Hard); 50-60% HRmax (~105-125 bpm) |

| **Monitoring** | Occasional breathlessness but comfortable |

**RPE Target:** 12-13

- "Somewhat Hard"

- Breathing noticeably deeper; conversation possible but slightly effortful

- This is considered the benchmark moderate intensity for cardiac rehabilitation

**Variety Options:**

- Monday/Wednesday/Friday: Continuous moderate exercise

- Tuesday/Thursday: 2 x 15-minute bouts with 5-minute rest between

\### Resistance Training

| Exercise | Sets | Reps | Progression |

|----------|------|------|-------------|

| Wall push-ups | 2 | 12-15 | Increase reps |

| Seated leg raises | 2 | 12-15 | Same |

| Seated bicep curls | 2 | 10-12 | Increase to 3-5 lb weights |

| Seated rows | 2 | 12-15 | Increase resistance |

| Seated marches | 2 | 12-15 | Same |

| Hip abduction | 2 | 12-15 each | Increase resistance |

| **NEW:** Seated shoulder press | 1-2 | 8-10 | 1-2 lb weights |

**Frequency:** 2-3 days/week (allow 48 hours between sessions for recovery)

\### Cool-Down (5-10 minutes)

Same; increase stretch duration to 30-45 seconds.

\### Flexibility

Same; hold stretches for 45 seconds.

---

\## Week 4: Consolidation and Independence (Days 22-30)

\### Key Focus

Solidify routine, achieve full session duration, build long-term habits.

\### Warm-Up (5-10 minutes)

Same; efficient but thorough.

\### Aerobic Exercise

| Component | Specification |

|-----------|---------------|

| **Frequency** | 4-5 days/week |

| **Type** | Preferred modalities; continue variety |

| **Duration** | 30-35 minutes (target ≥150 minutes/week) |

| **Intensity** | RPE 12-14 (Somewhat Hard to Hard); 55-65% HRmax |

| **Monitoring** | Occasional breathlessness; able to speak with effort |

**RPE Target:** 12-14

- Low end: "Somewhat Hard" (12-13)

- Occasional "Hard" (14) for short intervals

- Ideal weekly aerobic volume: ≥150 minutes moderate exercise

**Progression Options:**

- Add 2-3 minute "steady state" periods at RPE 13-14

- For cycling: increase resistance slightly while maintaining cadence

\### Resistance Training

| Exercise | Sets | Reps | Progression |

|----------|------|------|-------------|

| Wall push-ups | 2 | 15 | Increase depth of push-up |

| Seated leg raises | 2 | 15 | Add ankle weights (1-2 lb) |

| Seated bicep curls | 2-3 | 10-12 | 3-5 lb weights |

| Seated rows | 2-3 | 12-15 | Increase resistance |

| Seated marches | 2 | 15 | Add ankle weights |

| Hip abduction | 2 | 15 each | Increase resistance |

| Seated shoulder press | 2 | 10-12 | 2-3 lb weights |

**Frequency:** 2-3 days/week (48 hours between sessions)

\### Cool-Down (5-10 minutes)

Same; hold stretches 45-60 seconds.

\### Flexibility

Hold stretches 45-60 seconds; consider adding a gentle yoga-style routine.

---

\## Condition-Specific Modifications

\### For Preserved LVEF (55%)

- This LVEF is within normal range for a 60-year-old female

- Allows for more aggressive progression compared to patients with reduced EF

- Continue to monitor symptoms despite reassuring EF

\### For Patient Reluctance/Anxiety

- **Week 1 emphasis:** Reassurance that exercise is safe and beneficial

- **Use of HR monitoring:** Provides objective safety confirmation

- **Celebrate small wins:** Acknowledge completion of each session

- **Education:** Explain that RPE 9-11 is lower than pre-MI exercise levels; this is intentional for safety

- **Support:** Consider involving family member or friend for accountability

\### RPE and Heart Rate Correlation

| RPE Score | Perceived Exertion | Approx. %HRmax | Talk Test |

|-----------|-------------------|---------------|-----------|

| 9-11 | Very Light to Light | 40-50% | Conversation easy |

| 12-13 | Somewhat Hard | 50-60% | Conversation requires effort |

| 14 | Hard | 60-65% | Conversation short phrases |

| 15+ | Very Hard | >65% | NOT recommended |

\### Absolute Contraindications to Exercise

- Uncontrolled arrhythmias or tachycardia

- Uncontrolled hypertension (SBP >180 or DBP >100)

- Acute systemic illness or fever

- Unstable angina

- Uncompensated heart failure

---

\## Documentation and Monitoring

\### Daily Exercise Log

**Date:** _____ **RPE:** _____ **HR (if measured):** _____

**Exercise Session Record:**

| Session | Duration (min) | Average RPE | Peak RPE | Notes |

|---------|----------------|-------------|----------|-------|

| Week 1 | 15-20 | 9-10 | 11 | |

| Week 2 | 20-25 | 10-11 | 12 | |

| Week 3 | 25-30 | 11-12 | 13 | |

| Week 4 | 30-35 | 12-13 | 14 | |

\### Weekly Check-in Questions

1. Any chest pain or discomfort during/after exercise? ____

2. Any unusual shortness of breath? ____

3. Any dizziness or lightheadedness? ____

4. Level of confidence in exercise ability (1-10): ____

5. Enjoyment of activity (1-10): ____

---

\## Maintenance and Progression Beyond 30 Days

After completing the 30-day program, the patient should:

\### Continue

- Aerobic exercise ≥150 minutes/week at RPE 12-14

- Resistance training 2-3 sessions/week with 48-hour recovery

- Regular warm-up and cool-down routines

\### Progress

- Consider increasing resistance training to 3 sets of 10-15 reps

- Consider incorporating high-intensity interval training (HIIT) if cleared by physician

- Maintain variety to prevent boredom

\### Safety

- Continue using RPE monitoring

- Report any new symptoms to healthcare provider

- Periodic reassessment (every 3-6 months) recommended

---

\## Quick Reference Card

\### Borg RPE 6-20 Scale

| Score | Exertion Level | Target? |

|-------|----------------|---------|

| 6 | No exertion | Warm-up |

| 7-8 | Extremely Light | ✓ Week 1 |

| 9 | Very Light | ✓ Week 1-2 |

| 10 | | ✓ Week 2 |

| 11 | Light | ✓ Week 2-3 |

| 12 | | ✓ Week 3-4 |

| 13 | Somewhat Hard | ✓ Week 3-4 |

| 14 | | Limit |

| 15 | Hard | Avoid |

| 16-17 | Very Hard | Avoid |

| 18-19 | Extremely Hard | Avoid |

| 20 | Maximal exertion | Avoid |

\### Red Flags - STOP Exercise

- Chest pain/pressure

- Dizziness/nausea

- Racing/skipping heartbeat

- Unusual shortness of breath

- Arm/jaw/neck pain

*Always consult with the patient's cardiologist before beginning any exercise program and ensure appropriate medical clearance.*

**Patient 3**

**Atrial fibrillation (AF):** 65-year-old male, paroxysmal AF, rate-controlled on beta-blocker therapy, CHA₂DS₂-VASc score 3, no significant structural heart disease, LVEF 60%.

\# 30-Day Cardiac Rehabilitation Exercise Program

\## Patient: 65-year-old Male with Paroxysmal Atrial Fibrillation

---

\## Patient Profile Summary

| Characteristic | Details |

|----------------|---------|

| Age | 65 years |

| Diagnosis | Paroxysmal Atrial Fibrillation (AF) |

| Rate Control | Beta-blocker therapy |

| CHA₂DS₂-VASc Score | 3 |

| Structural Heart Disease | None |

| LVEF | 60% (preserved) |

---

\## Critical Safety Considerations for AF Patients on Beta-Blockers

\### Heart Rate Limitations

Patients on beta-blocker therapy have a blunted heart rate response to exercise. **Predicted maximum heart rate formulas (e.g., 220 - age) are unreliable** for this population. Studies demonstrate that target heart rate values computed using predicted HRmax seldom result in appropriate prescribed exercise intensity for patients on beta-blockers.

**Recommended Approach:**

- **Do NOT use %HRmax** for intensity prescription

- **Use Borg RPE (6-20 scale) as the primary intensity guide**

- Target RPE: **11-13 ("fairly light" to "somewhat hard")** for moderate-intensity exercise

- Use the "Talk Test": patient should be able to speak in short sentences but not sing

\### Important Caveat for AF Patients

Research in AF patients shows that **Borg RPE 13 does not consistently coincide with the ventilatory anaerobic threshold** in this population. In a study of 150 AF patients post-ablation, the workload at Borg RPE 13 was significantly higher than the workload at the ventilatory threshold (67.2W vs 54.7W). This means:

> **RPE should be used cautiously and conservatively in AF patients. Start at the lower end of the target range (RPE 9-11) and progress only with medical supervision.**

\### Resting Heart Rate Targets

Based on guideline recommendations for rate-controlled AF:

| Parameter | Target |

|-----------|--------|

| Resting HR (symptomatic) | <80 bpm |

| Resting HR (asymptomatic) | <110 bpm |

| Exercise HR | Conservative approach recommended |

---

\## FITT-VP Framework

| Component | Recommendation |

|-----------|----------------|

| **Frequency** | 3-5 days/week aerobic; 2-3 days/week resistance |

| **Intensity** | RPE 9-13 (Borg 6-20 scale) |

| **Time** | 20-40 minutes aerobic; 20-30 minutes resistance + flexibility |

| **Type** | Continuous aerobic + circuit resistance + flexibility |

| **Volume** | Gradual progression to 150 min/week moderate intensity |

| **Progression** | RPE and duration-based, NOT heart rate-based |

---

\# 30-Day Program: Weekly Breakdown

---

\## Week 1 (Days 1-7): Foundation and Assessment Phase

\### Goals:

- Establish baseline exercise tolerance

- Ensure safe response to exercise

- Build patient confidence

- Monitor for AF symptoms during activity

\### Daily Structure (Total: 30-35 minutes per session)

| Phase | Duration | Activity |

|-------|----------|----------|

| Warm-up | 5-7 min | Light walking, arm circles, ankle circles, gentle trunk rotation |

| Aerobic | 10-15 min | Continuous walking (treadmill or track) OR Recumbent stationary bike |

| Resistance | 10 min | Bodyweight exercises only (see below) |

| Cool-down | 5 min | Slow walking, gentle stretching |

| Flexibility | 5 min | Static stretching (major muscle groups) |

\### Aerobic Exercise Details

| Parameter | Prescription |

|-----------|--------------|

| Type | Treadmill walking OR Recumbent stationary bike |

| Duration | 10-15 minutes continuous |

| Intensity Target | **RPE 9-11** ("very light" to "fairly light") |

| Frequency | 3 days/week (e.g., Monday, Wednesday, Friday) |

\### Resistance Training Details

| Exercise | Sets | Reps | Rest |

|----------|------|------|------|

| Wall push-ups | 1 | 10 | 60 sec |

| Seated arm curls (light dumbbells 1-2 kg) | 1 | 10 | 60 sec |

| Seated knee extensions | 1 | 10 | 60 sec |

| Seated shoulder press (light dumbbells 1-2 kg) | 1 | 10 | 60 sec |

**Resistance Training Frequency:** 2 days/week (e.g., Tuesday, Thursday)

\### Flexibility Exercises (Hold each 15-20 seconds)

- Standing calf stretch

- Seated hamstring stretch

- Chest stretch (doorway)

- Upper trapezius stretch

- Triceps stretch

\### Precautions - Week 1:

- Perform all exercises in a supervised setting if possible

- Monitor for palpitations, dizziness, or chest discomfort

- Check pulse before and after exercise (document)

- If RPE exceeds 11, reduce intensity immediately

- Do not exercise if resting HR >100 bpm or if in AF episode

---

\## Week 2 (Days 8-14): Early Progression Phase

\### Goals:

- Increase exercise duration

- Maintain appropriate intensity

- Introduce light resistance progression

- Establish consistent exercise routine

\### Daily Structure (Total: 35-40 minutes per session)

| Phase | Duration | Activity |

|-------|----------|----------|

| Warm-up | 5-7 min | Light walking, dynamic stretches |

| Aerobic | 15-20 min | Walking OR Stationary bike |

| Resistance | 10-12 min | Bodyweight + Light weights |

| Cool-down | 5 min | Slow walking |

| Flexibility | 5 min | Static stretching |

\### Aerobic Exercise Details

| Parameter | Prescription |

|-----------|--------------|

| Type | Treadmill walking OR Recumbent stationary bike |

| Duration | 15-20 minutes continuous |

| Intensity Target | **RPE 11-12** ("fairly light" to "somewhat hard") |

| Frequency | 3-4 days/week |

\### Resistance Training (Progression)

**Frequency:** 2-3 days/week (48 hours between sessions)

| Exercise | Sets | Reps | Rest |

|----------|------|------|------|

| Wall push-ups | 2 | 10 | 60 sec |

| Seated arm curls (2-3 kg) | 2 | 10 | 60 sec |

| Seated knee extensions (ankle weights 1 kg) | 2 | 10 | 60 sec |

| Seated shoulder press (2-3 kg) | 2 | 10 | 60 sec |

| Standing heel raises | 2 | 12 | 60 sec |

\### Precautions - Week 2:

- Monitor RPE closely; do not exceed 12

- Check for delayed onset muscle soreness

- If soreness >2/10, reduce resistance training frequency

- Maintain hydration before and after exercise

- Continue pulse monitoring pre- and post-exercise

---

\## Week 3 (Days 15-21): Consolidation Phase

\### Goals:

- Increase aerobic duration to 25 minutes

- Maintain RPE targets with improved workload

- Progressive resistance training

- Build exercise self-efficacy

\### Daily Structure (Total: 40-45 minutes per session)

| Phase | Duration | Activity |

|-------|----------|----------|

| Warm-up | 5-10 min | Light walking, dynamic stretches, arm swings |

| Aerobic | 20-25 min | Walking OR Stationary bike OR Combined |

| Resistance | 12-15 min | Progressive resistance |

| Cool-down | 5 min | Slow walking, gentle stretching |

| Flexibility | 5-10 min | Static stretching (emphasis on tight muscles) |

\### Aerobic Exercise Details

| Parameter | Prescription |

|-----------|--------------|

| Type | Treadmill walking OR Stationary bike OR Pool walking (if available) |

| Duration | 20-25 minutes continuous |

| Intensity Target | **RPE 11-13** ("fairly light" to "somewhat hard") |

| Frequency | 4 days/week |

\### Resistance Training (Progression)

**Frequency:** 2-3 days/week (48 hours between sessions)

| Exercise | Sets | Reps | Rest |

|----------|------|------|------|

| Wall push-ups (progress to counter push-ups if tolerated) | 2-3 | 10-12 | 60 sec |

| Seated arm curls (3 kg) | 2-3 | 10-12 | 60 sec |

| Seated knee extensions (ankle weights 1-2 kg) | 2-3 | 10-12 | 60 sec |

| Seated shoulder press (2-3 kg) | 2-3 | 10-12 | 60 sec |

| Standing heel raises (add light weights if tolerated) | 2 | 12-15 | 60 sec |

| Seated rows (Theraband) | 2 | 12 | 60 sec |

\### Precautions - Week 3:

- Progress duration before increasing intensity

- If AF episode occurs, rest and resume at lower RPE

- Consider exercise in cooler environments to reduce cardiac stress

- Monitor for signs of overtraining (fatigue, resting HR >90 bpm)

- Continue RPE-based intensity prescription

---

\## Week 4 (Days 22-30): Maintenance and Transition Phase

\### Goals:

- Achieve 30 minutes of continuous aerobic exercise

- Maintain RPE 12-13 for majority of session

- Resistance training at moderate intensity

- Prepare for ongoing independent exercise program

\### Daily Structure (Total: 45-50 minutes per session)

| Phase | Duration | Activity |

|-------|----------|----------|

| Warm-up | 5-10 min | Light walking, dynamic stretches, joint mobilization |

| Aerobic | 25-30 min | Walking OR Stationary bike OR Combined |

| Resistance | 12-15 min | Progressive resistance |

| Cool-down | 5 min | Slow walking, gentle stretching |

| Flexibility | 5-10 min | Full body static stretching |

\### Aerobic Exercise Details

| Parameter | Prescription |

|-----------|--------------|

| Type | Treadmill walking OR Stationary bike OR Outdoor walking (if safe) |

| Duration | 25-30 minutes continuous |

| Intensity Target | **RPE 12-13** ("somewhat hard") |

| Frequency | 4-5 days/week |

\### Resistance Training (Final Progression)

**Frequency:** 2-3 days/week (48 hours between sessions)

| Exercise | Sets | Reps | Rest |

|----------|------|------|------|

| Wall push-ups (progress to counter push-ups) | 3 | 12 | 60 sec |

| Seated arm curls (3-4 kg) | 3 | 12 | 60 sec |

| Seated knee extensions (ankle weights 2 kg) | 3 | 12 | 60 sec |

| Seated shoulder press (3 kg) | 3 | 12 | 60 sec |

| Standing heel raises (with weights) | 3 | 15 | 60 sec |

| Seated rows (Theraband, increased tension) | 3 | 12 | 60 sec |

| Modified squats (chair-assisted) | 2 | 10 | 60 sec |

\### Precautions - Week 4:

- Complete a full 30-day assessment of progress

- Document resting HR, exercise HR, RPE, and any AF symptoms

- If RPE consistently <11, consider mild intensity increase

- Continue to avoid maximal exertion (RPE >14)

- Plan for ongoing maintenance program

---

\## Weekly Summary Table

| Week | Aerobic Duration | Aerobic Frequency | Aerobic RPE Target | Resistance Frequency | Resistance Intensity |

|------|------------------|-------------------|--------------------|---------------------|---------------------|

| 1 | 10-15 min | 3 days | 9-11 | 2 days | Bodyweight only |

| 2 | 15-20 min | 3-4 days | 11-12 | 2-3 days | Light (1-3 kg) |

| 3 | 20-25 min | 4 days | 11-13 | 2-3 days | Light-Moderate |

| 4 | 25-30 min | 4-5 days | 12-13 | 2-3 days | Moderate |

---

\## Condition-Specific Modifications for Atrial Fibrillation

\### Exercise During AF Episodes

| Situation | Action |

|-----------|--------|

| **Asymptomatic AF episode** | Continue with reduced intensity (RPE 9-10); monitor symptoms |

| **Symptomatic AF (palpitations, dyspnea, dizziness)** | STOP exercise immediately; rest until symptoms resolve |

| **Resting HR >110 bpm before exercise** | Defer exercise session; reassess in 1-2 hours |

| **Chest pain or severe dyspnea** | STOP; seek medical attention if persistent |

\### Comorbidity Management (ESC 2024 AF Guidelines)

The updated ESC guidelines emphasize that **comorbidity management is central to AF care** and should be prioritized alongside rhythm control. For this patient with CHA₂DS₂-VASc score of 3:

- **Anticoagulation:** Appropriate OAC therapy is essential (Class I recommendation)

- **Hypertension control:** Target BP <130/80 mmHg

- **Weight management:** If BMI >25, weight loss of ≥10% recommended

- **Alcohol reduction:** Limit to ≤2 units/day

- **Sleep apnea screening:** Consider if symptomatic

\### Beta-Blocker Exercise Considerations

| Issue | Management Strategy |

|-------|---------------------|

| Blunted heart rate response | Use RPE, NOT heart rate for intensity prescription |

| Exercise fatigue | May be more prominent initially; expect adaptation over 2-3 weeks |

| Post-exercise recovery | Allow extended cool-down (5-10 minutes minimum) |

| Medication timing | Exercise 1-2 hours after beta-blocker dose if possible |

---

\## Exercise Prescription Parameters Summary (Based on CSANZ Guidelines)

According to the Cardiac Society of Australia and New Zealand position statement:

| Parameter | This Patient's Prescription |

|-----------|----------------------------|

| **Aerobic intensity** | 55-70% HRmax equivalent (but RPE-based) |

| **Aerobic RPE target** | 11-13 (Borg 6-20 scale) |

| **Resistance intensity** | 50-70% of 1RM (moderate) |

| **Resistance RPE target** | 5-7 (Borg 6-20 scale) |

| **Resistance tempo** | >4 seconds per repetition (>1 sec concentric, >3 sec eccentric) |

| **Rest between sets** | 60 seconds |

| **Weekly volume target** | 150 minutes moderate intensity (by Week 4) |

---

\## Warning Signs: When to STOP Exercise Immediately

**STOP if any of the following occur:**

- Chest pain, pressure, or tightness

- Dizziness, lightheadedness, or near-syncope

- Palpitations or irregular heartbeat that feels different from usual AF

- Shortness of breath disproportionate to effort

- Nausea or cold sweats

- RPE >14 ("hard" or higher)

- Confusion or disorientation

**If any of these occur:**

1. Stop exercise immediately

2. Sit or lie down in a comfortable position

3. Monitor symptoms

4. If symptoms persist >5 minutes, seek medical attention

5. Notify cardiologist before next session

---

\## Daily Session Checklist

- [ ] Check resting HR (should be <100 bpm)

- [ ] Take prescribed medications as scheduled

- [ ] Warm-up completed (5-10 minutes)

- [ ] Aerobic session performed at target RPE

- [ ] Resistance training performed with proper form

- [ ] Cool-down completed (5 minutes)

- [ ] Flexibility exercises performed

- [ ] Post-exercise HR and RPE documented

- [ ] AF symptoms or palpitations noted

- [ ] Hydration maintained

---

\## Notes on Progression

1. **Intensity progression** should be based on RPE, NOT heart rate

2. **Duration progression** should precede intensity progression

3. **Frequency progression** from 3 to 5 days/week as tolerated

4. **Resistance progression** should follow the "2-for-2 rule": if patient can complete 2 additional reps above target for 2 consecutive sessions, increase weight by 0.5-1 kg

5. **Progression should be paused** during any AF episodes or illness

---

\## References

1. Keteyian SJ, et al. Among Patients Taking Beta-Adrenergic Blockade Therapy, Use Measured (Not Predicted) Maximal Heart Rate to Calculate a Target Heart Rate for Cardiac Rehabilitation. *J Cardiopulm Rehabil Prev*. 2023

2. Van Gelder IC, et al. 2024 ESC Guidelines for the Management of Atrial Fibrillation. *Eur Heart J*. 2024

3. Kusachi S, et al. Borg's exertion scale did not coincide with ventilatory anaerobic threshold in atrial fibrillation patients. *J Exerc Rehabil*. 2024

4. CSANZ Position Statement on Exercise and Physical Activity in Cardiac Rehabilitation. 2023

5. LeWine HE. Do beta blockers interfere with exercise? *Harvard Health Publishing*. 2024

6. ACCF/AHA/HRS Guidelines for Atrial Fibrillation Management - Target Rates Table

---

*This program is designed as a template for medical professionals. Individual patient response to exercise should be monitored and the program adjusted accordingly. All exercise should be performed under appropriate medical supervision for patients with atrial fibrillation.*

**Patient 4**

Heart Failure with Reduced Ejection Fraction (HFrEF): 70-year-old female, ischemic cardiomyopathy, LVEF 35%, New York Heart Association (NYHA) Class II symptoms, stable on guideline-directed medical therapy**.**

\# 30-Day Cardiac Rehabilitation Exercise Program for HFrEF Patient

\## Patient Profile Summary

- **Age/Sex:** 70-year-old female

- **Condition:** Ischemic cardiomyopathy, HFrEF

- **LVEF:** 35%

- **NYHA Class:** II (mild symptoms with ordinary activity)

- **Medication Status:** Stable on guideline-directed medical therapy (GDMT)

---

\## Program Foundation & Safety Principles

\### Clinical Guidelines Reference

This program adheres to AHA/ACC/HFSA and ESC Class I recommendations for exercise training in stable HFrEF patients to improve functional capacity, quality of life, and reduce hospitalization risk . The FITT-VP framework (Frequency, Intensity, Time, Type, Volume, Progression) is applied throughout.

\### Universal Safety Precautions

1. **Medical Clearance:** Verify physician approval prior to program initiation

2. **Pre-exercise Assessment:** Monitor resting HR, BP, O₂ saturation, and symptom status

3. **Session Termination Criteria:** Stop immediately if experiencing:

- Chest pain/pressure

- Dizziness or near-syncope

- Excessive dyspnea (cannot speak in short sentences)

- RPE exceeding prescribed target

- Systolic BP drop >20 mmHg with exercise

- Irregular rhythm/palpitations

4. **Medication Considerations:** Beta-blockers may blunt HR response—**RPE is the primary intensity monitor**

---

\## Week 1: Foundation & Adaptation Phase

\### Frequency

- **Aerobic:** 3 days/week (non-consecutive days, e.g., Mon/Wed/Fri)

- **Resistance:** 2 days/week (Tue/Thu) – separated from aerobic sessions or performed after aerobic with adequate rest

- **Flexibility:** Daily (following each session and on rest days)

\### Session Structure (Every session)

\#### Warm-up (10 minutes)

- **Mode:** Slow walking or stationary cycling with no resistance

- **Intensity:** RPE 9-10 (very light), 30-40% HRR

- **Sequence:**

- 5 minutes: Large muscle group rhythmic movement (walking or cycling)

- 3 minutes: Gentle arm circles and shoulder rolls (seated)

- 2 minutes: Ankle pumps and gentle trunk rotations (seated)

- **Monitoring:** Check BP and symptoms before transitioning to aerobic phase

---

\### Aerobic Exercise

\#### Type

- **Primary:** Stationary recumbent cycle ergometer (preferred for safety and hemodynamic monitoring)

- **Alternative:** Treadmill walking (if cycle unavailable)

\#### Intensity

- **RPE Target:** **11-13 (Fairly Light to Somewhat Hard)** on Borg 6-20 scale

- **%HRmax Target:** 40-60% HRmax

- **Monitoring:** RPE is primary due to beta-blocker use; HR is secondary

\#### Duration

- **Weeks 1:** 15-20 minutes continuous (or interval format: 5-minute bouts with 2-minute rest)

\#### Progression (Weeks 1)

| Week | Session 1 | Session 2 | Session 3 |

|------|-----------|-----------|-----------|

| Week 1 | 15 min | 15 min | 20 min |

| Week 2 | 20 min | 20 min | 25 min |

| Week 3 | 25 min | 25 min | 30 min |

| Week 4 | 30 min | 30 min | 35 min |

---

\### Resistance Training

\#### Type

- **Mode:** Seated exercises focusing on large muscle groups

- **Exercises:** Seated leg extensions, seated leg curls, seated chest press (light), seated rows, bicep curls, tricep extensions

\#### Initial Prescription

- **Intensity:** 40% of estimated 1RM

- **Sets:** 1 set per exercise (2 weeks), progress to 2 sets (weeks 3-4)

- **Repetitions:** 10-15 repetitions per set

- **Rest:** 1-2 minutes between exercises

- **Frequency:** 2 days/week (Tue/Thu)

\#### Safety Notes

- **Avoid:** Valsalva maneuver (exhale on effort, inhale on release)

- **Start:** No resistance (body weight) first session, add light resistance as tolerated

- **Monitor:** BP during and after each set; avoid >180/105 mmHg

---

\### Cool-down (10 minutes)

\#### Purpose

- **Mode:** Slow walking or cycling with zero resistance

- **Intensity:** RPE 9-10 (decrease progressively)

- **Duration:** 5 minutes active recovery followed by 5 minutes stretching

- **Monitoring:** Check HR and BP at rest after cool-down

---

\### Flexibility Training

\#### Mode

- **Post-session:** Static stretching of major muscle groups

- **Exercises:** Hamstring stretch (seated), quadriceps stretch (standing, supported), calf stretch, chest stretch (doorway), upper back stretch

- **Duration:** 3-4 reps per muscle group, hold 20-30 seconds

- **Intensity:** Mild tension, no pain

- **Frequency:** Daily

---

\## Weeks 2-4: Progression Phase

\### Aerobic Progression

**Weeks 2:**

- Frequency: 3 days/week

- Duration: 20-25 minutes

- Intensity: RPE 11-13 (maintain)

- Consider: Begin 30-second interval increases if tolerated

**Weeks 3:**

- Frequency: 3-4 days/week

- Duration: 25-30 minutes

- Intensity: RPE 11-14 (low end of moderate)

- Consider: Add 1 minute to each session

**Weeks 4:**

- Frequency: 4 days/week

- Duration: 30-35 minutes

- Intensity: RPE 12-14 (Somewhat Hard) for portions

- Consider: Start interval format (2-3 minutes moderate, 1 minute light)

---

\### Resistance Progression

**Weeks 2:**

- Increase to 2 sets per exercise

- Maintain 10-15 reps at 40% 1RM

- Add seated calf raises to exercise list

**Weeks 3:**

- Progress to 40-50% 1RM

- 2 sets, 10-12 reps

- Maintain 1-2 min rest

- Consider: Add one new exercise (seated shoulder press)

**Weeks 4:**

- 40-50% 1RM, 2-3 sets, 10-12 reps

- Maintain 1-2 min rest

- Consider: Circuit format (alternate upper/lower body) for efficiency

---

\## Detailed 30-Day Schedule

\### Days 1-7 (Week 1)

| Day | Activity | Duration | Intensity | Notes |

|-----|----------|----------|-----------|-------|

| Day 1 (Mon) | Aerobic + Flex | 15 min | RPE 11-12 | Stationary cycle |

| Day 2 (Tue) | Resistance + Flex | 1 set x 10-15 reps | 40% 1RM | Seated exercises |

| Day 3 (Wed) | Aerobic + Flex | 15 min | RPE 11-12 | Stationary cycle |

| Day 4 (Thu) | Resistance + Flex | 1 set x 10-15 reps | 40% 1RM | Seated exercises |

| Day 5 (Fri) | Aerobic + Flex | 20 min | RPE 11-12 | Stationary cycle |

| Day 6 (Sat) | Flexibility only | 15 min | Light | Active recovery |

| Day 7 (Sun) | Rest | - | - | - |

\### Days 8-14 (Week 2)

| Day | Activity | Duration | Intensity | Notes |

|-----|----------|----------|-----------|-------|

| Day 8 (Mon) | Aerobic + Flex | 20 min | RPE 11-13 | Cycle/walk |

| Day 9 (Tue) | Resistance + Flex | 2 sets x 10-15 reps | 40% 1RM | Add calf raises |

| Day 10 (Wed) | Aerobic + Flex | 20 min | RPE 11-13 | Cycle/walk |

| Day 11 (Thu) | Resistance + Flex | 2 sets x 10-15 reps | 40% 1RM | All exercises |

| Day 12 (Fri) | Aerobic + Flex | 25 min | RPE 11-13 | Cycle/walk |

| Day 13 (Sat) | Flexibility only | 15 min | Light | Active recovery |

| Day 14 (Sun) | Rest | - | - | - |

\### Days 15-21 (Week 3)

| Day | Activity | Duration | Intensity | Notes |

|-----|----------|----------|-----------|-------|

| Day 15 (Mon) | Aerobic + Flex | 25 min | RPE 11-14 | Cycle/walk |

| Day 16 (Tue) | Resistance + Flex | 2 sets x 10-12 reps | 40-50% 1RM | All exercises |

| Day 17 (Wed) | Aerobic + Flex | 25 min | RPE 11-14 | Add intervals |

| Day 18 (Thu) | Resistance + Flex | 2 sets x 10-12 reps | 40-50% 1RM | Add shoulder press |

| Day 19 (Fri) | Aerobic + Flex | 30 min | RPE 11-14 | Cycle/walk |

| Day 20 (Sat) | Flexibility only | 15 min | Light | Active recovery |

| Day 21 (Sun) | Rest | - | - | - |

\### Days 22-30 (Week 4)

| Day | Activity | Duration | Intensity | Notes |

|-----|----------|----------|-----------|-------|

| Day 22 (Mon) | Aerobic + Flex | 30 min | RPE 12-14 | Cycle/walk |

| Day 23 (Tue) | Resistance + Flex | 2-3 sets x 10-12 reps | 40-50% 1RM | Circuit format |

| Day 24 (Wed) | Aerobic + Flex | 30 min | RPE 12-14 | Intervals |

| Day 25 (Thu) | Resistance + Flex | 2-3 sets x 10-12 reps | 40-50% 1RM | Circuit format |

| Day 26 (Fri) | Aerobic + Flex | 35 min | RPE 12-14 | Intervals |

| Day 27 (Sat) | Aerobic + Flex | 25 min | RPE 11-13 | Optional extra |

| Day 28 (Sun) | Rest | - | - | - |

| Day 29 (Mon) | Aerobic + Flex | 35 min | RPE 12-14 | Peak session |

| Day 30 (Tue) | Resistance + Flex | 2-3 sets | 40-50% 1RM | Final assessment |

---

\## Condition-Specific Modifications & Precautions

\### HFrEF Considerations

**NYHA Class II Specific:**

- Patients with mild symptoms may exercise with supervision

- Monitor for symptom progression (dyspnea, fatigue)

- Begin with 15-minute sessions; these patients often tolerate up to 40-60 minutes with proper warm-up

**Medication Effects:**

- Beta-blockers blunt HR response—**RPE is more reliable**

- Diuretics may cause electrolyte imbalances; ensure hydration

- ACE inhibitors/ARBs may cause BP drops; monitor postural changes

**Ischemic Cardiomyopathy:**

- Monitor for angina equivalents (dyspnea, fatigue, arm/shoulder discomfort)

- Keep RPE at 11-14 to avoid ischemic threshold

- Ensure pre-exercise BP < 180/105 and > 90/60

---

\### Progression Signs & Safety Triggers

**Safe to Progress if:**

- RPE during last 3 sessions ≤ target range

- No symptoms during or after exercise

- HR recovery: >12 bpm drop in 1 minute post-exercise

- Stable body weight (no fluid retention signs)

**Hold/Reduce if:**

- Weight gain >2 pounds in 24 hours or >5 pounds in 1 week

- Increased dyspnea, orthopnea, or paroxysmal nocturnal dyspnea

- RPE >15 (Hard) at current workload

- BP >180/105 or <90/60 at rest

- Any angina, dizziness, or palpitations

- Fatigue or weakness requiring rest >2 hours after exercise

**Discontinue and Contact Physician if:**

- Chest pain/pressure

- Significant dizziness or near-syncope

- Severe dyspnea at rest or with minimal effort

- Irregular heart rhythm

- Prolonged recovery symptoms (>20 minutes)

---

\## Outcome Assessment Timeline

| Time Point | Assessments |

|------------|-------------|

| Baseline | 6-Minute Walk Test (6MWT), RPE rating during exercise, resting HR/BP, NYHA class, MLHFQ (quality of life) |

| Day 15 (Midpoint) | 6MWT (optional), symptom review, RPE calibration check |

| Day 30 (Completion) | 6MWT, resting HR/BP, RPE rating, MLHFQ, exercise duration/volume achieved, physician consultation for next phase |

---

\## Additional Recommendations

\### Inspiratory Muscle Training (IMT)

- **Indication:** Patients with reduced inspiratory muscle strength (PI-max ≤70% predicted), ventilatory limitation, or dyspnea symptoms

- **Prescription:** 20-40% of PI-max, 2 x 15 minutes/day, 3-4 times/week

- **Timing:** Can be added from week 2 if indicated

\### Patient Education Points

1. **Symptom Awareness:** Recognize early warning signs of decompensation

2. **Fluid Balance:** Monitor daily weight; report changes

3. **Hydration:** Drink small amounts during exercise (not excessive)

4. **Pacing:** Break activities into shorter bouts if fatigued

5. **Medication Adherence:** Continue GDMT as prescribed

---

\## References

1. Exercise training in HFrEF improves functional capacity, quality of life, and reduces hospitalizations (AHA/ACC/HFSA Class I, ESC Class I)

2. RPE Borg 11-14 target for aerobic exercise in HF patients

3. Resistance training at 40-50% 1RM, 2-3 sets, 10-15 reps recommended

4. Warm-up and cool-down at RPE 9-11, 10 minutes each

5. Inspiratory muscle training adjunct for patients with reduced respiratory muscle strength

**Patient 5**

Heart Failure with Preserved Ejection Fraction (HFpEF): 68-year-old male, history of hypertension and diabetes, LVEF 50%, evidence of diastolic dysfunction, NYHA Class II symptoms (predominantly dyspnea on exertion).

\# 30-Day Cardiac Rehabilitation Exercise Program

\## Patient: 68-year-old Male with HFpEF, NYHA Class II

---

\## I. Patient Profile & Baseline Considerations

| Characteristic | Details |

|---------------|---------|

| Age | 68 years |

| Sex | Male |

| Primary Diagnosis | Heart Failure with Preserved Ejection Fraction (HFpEF) |

| LVEF | 50% |

| Key Comorbidities | Hypertension, Type 2 Diabetes |

| Functional Class | NYHA Class II (dyspnea on exertion) |

| Primary Limitation | Diastolic dysfunction with impaired ventricular filling |

\### Critical Safety Precautions

**Pre-exercise Screening Required Before Each Session:**

- Resting heart rate between 50-100 bpm

- Systolic BP > 80 mmHg

- No evidence of congestion or volume overload

- Stable symptoms for ≥ 2 weeks prior to program initiation

- No resting symptoms or postural hypotension

**Absolute Contraindications to Exercise:**

- Uncontrolled arrhythmias

- Acute systemic illness or fever

- Unstable angina

- Uncontrolled hypertension (SBP > 180 mmHg or DBP > 110 mmHg)

- Significant fluid overload or pulmonary congestion

**Symptom-Limited Exercise Testing Consideration:**

Current evidence suggests that maximal exercise testing may cause sustained impairment of diastolic function in patients with significant cardiac impairment. For this patient, submaximal testing or cautious ramp protocols with close symptom monitoring are recommended over maximal effort testing .

---

\## II. Program Design (FITT-VP Framework)

\### A. Warm-Up (5-10 minutes) - **Every Session**

| Component | Details |

|-----------|---------|

| **Type** | Low-intensity rhythmic movements using large muscle groups |

| **Activities** | • Seated marching or slow stationary cycling (no resistance)<br>• Arm circles and shoulder rolls<br>• Gentle trunk rotations<br>• Ankle pumps |

| **Intensity** | RPE 6-8 (very light) - No increase in dyspnea |

| **Duration** | 5-10 minutes |

| **Purpose** | Gradual cardiovascular adaptation, prepare musculoskeletal system |

---

\### B. Aerobic Exercise

**Weeks 1-2 (Foundation Phase)**

| Parameter | Prescription |

|-----------|-------------|

| **Type** | • Stationary cycle ergometry (preferred for workload precision)<br>• Treadmill walking<br>• Recumbent stepper (if available) |

| **Frequency** | 3 sessions/week (non-consecutive days: e.g., Mon/Wed/Fri) |

| **Intensity** | RPE 9-11 (very light to fairly light) - **30-40% HRR** |

| **Duration** | 10-15 minutes continuous OR two 5-7 minute bouts with 2-3 minute rest between |

| **Safety Focus** | Maintain ability to speak in full sentences. Monitor for dyspnea, dizziness, or chest discomfort. |

**Weeks 3-4 (Progression Phase)**

| Parameter | Prescription |

|-----------|-------------|

| **Type** | Same as weeks 1-2 |

| **Frequency** | 4 sessions/week |

| **Intensity** | RPE 11-13 (fairly light to somewhat hard) - **40-50% HRR** |

| **Duration** | 20-25 minutes continuous |

**Progression Protocol:**

- Increase duration before increasing intensity

- Add 2-3 minutes per session per week as tolerated

- If symptoms (dyspnea, fatigue) occur, return to previous duration for 2 sessions before attempting progression again

**Target Heart Rate Calculation:**

- Estimated HRmax = 220 - 68 = 152 bpm

- **Weeks 1-2:** 40-50% HRR → approximately 96-106 bpm (using resting HR ~70)

- **Weeks 3-4:** 50-60% HRR → approximately 106-116 bpm

- *Note: Beta-blockers may blunt HR response; emphasize RPE monitoring*

**RPE Borg 6-20 Scale Guidance:**

| Zone | RPE | Target for This Patient |

|------|-----|------------------------|

| **Cool-down** | 6-8 | ✓ |

| **Warm-up** | 6-8 | ✓ |

| **Training Zone - Week 1-2** | 9-11 | ✓ |

| **Training Zone - Week 3-4** | 11-13 | ✓ |

| **Above Target** | 14+ | ✗ STOP and reduce intensity |

| **Maximum** | 18-20 | ✗ Never target |

*Clinical Note: Studies demonstrate HFpEF patients achieve similar peak RPE values (15.9 ± 0.4) to healthy controls during exercise testing, indicating intact perception of exertion despite reduced functional capacity .*

---

\### C. Resistance Training

**General Principles:**

- Start **Week 2** only after aerobic tolerance is established

- Perform on non-consecutive days (e.g., Tue/Thu)

- **Weeks 1-2:** Bodyweight and light resistance bands only

**Prescription (Weeks 2-4):**

| Parameter | Details |

|-----------|---------|

| **Frequency** | 2 sessions/week (non-consecutive days) |

| **Intensity** | 40-50% 1RM (very light to light) |

| **Sets** | 1-2 sets per exercise |

| **Repetitions** | 10-15 repetitions |

| **Rest Between Sets** | 60-90 seconds |

| **RPE Target** | 11-13 (somewhat hard) on Borg 6-20 scale |

**Exercise Selection:**

| Exercise | Muscle Group | Progression Notes |

|----------|--------------|-------------------|

| Seated leg extensions | Quadriceps | Start: bodyweight; Progress: ankle weights 1-2 lbs |

| Seated hamstring curls | Hamstrings | As above |

| Wall sits (30-45 sec) | Lower body | Monitor for breath-holding |

| Seated rows with resistance band | Upper back | Start: light band only |

| Chest press with bands | Pectorals | Maintain stable seated position |

| Bicep curls (light weights) | Arms | 1-3 lb dumbbells initial |

| Standing heel raises | Calves | Hold onto chair for balance |

**Critical Resistance Training Safety Rules:**

1. **Never hold your breath** during lifting (Valsalva maneuver increases intrathoracic pressure and can impair venous return)

2. Exhale during exertion (lifting phase); inhale during lowering phase

3. Maintain controlled tempo: 2-second concentric, 3-second eccentric

4. Stop immediately if chest pain, severe dyspnea, dizziness, or palpitations occur

**Resistance Training Progression (Weeks 3-4):**

- Increase repetitions from 10 to 15 before increasing resistance

- Once 15 repetitions can be completed with RPE ≤13, increase resistance by 5-10%

- Add second set when patient tolerates 15 reps × 1 set with RPE 12-13

---

\### D. Cool-Down (5-10 minutes) - **Every Session**

| Component | Details |

|-----------|---------|

| **Type** | Low-intensity aerobic activity + flexibility |

| **Activities** | • Slow walking or unloaded cycling (2-3 min)<br>• Gradual reduction of pace |

| **Intensity** | RPE 6-8 |

| **Flexibility** | Static stretching (hold 15-30 seconds, 2-3 reps each) |

| **Key Stretches** | • Standing calf stretch<br>• Seated hamstring stretch<br>• Chest stretch (doorway)<br>• Quadriceps stretch (standing, holding support) |

**Cool-Down Safety:**

- **Never** stop abruptly; this can cause blood pooling and orthostatic hypotension

- Monitor for dizziness during transition from exercise to rest

- Encourage slow, deep breathing during stretches

---

\## III. 30-Day Weekly Progression Schedule

\### Week 1: Initiation Phase

**Focus:** Establishing baseline tolerance, learning RPE monitoring, safety education

| Day | Session Type | Aerobic | Resistance | Total Time |

|-----|-------------|---------|------------|------------|

| Monday | Aerobic | 10 min cycling/walking @ RPE 9-11 | - | ~15 min |

| Tuesday | Rest | - | - | - |

| Wednesday | Aerobic | 12 min @ RPE 9-11 | - | ~17 min |

| Thursday | Rest | - | - | - |

| Friday | Aerobic | 10-12 min @ RPE 9-11 | - | ~15-17 min |

| Weekend | Active Rest | Light walking (5-10 min, RPE ≤8) | - | - |

**Key Milestone Week 1:** Successfully complete 3 aerobic sessions without symptom exacerbation. Teach patient to identify "early warning signs" of overexertion.

---

\### Week 2: Introduction of Resistance Training

**Focus:** Adding controlled strength work, maintaining aerobic progress

| Day | Session Type | Aerobic | Resistance | Total Time |

|-----|-------------|---------|------------|------------|

| Monday | Aerobic | 15 min @ RPE 10-12 | - | ~20 min |

| Tuesday | Combined | 10 min @ RPE 9-11 | 1 set × 10 reps, 6 exercises @ RPE 11 | ~30 min |

| Wednesday | Aerobic | 15-18 min @ RPE 10-12 | - | ~22 min |

| Thursday | Rest | - | - | - |

| Friday | Combined | 10-12 min @ RPE 9-11 | 1 set × 10-12 reps, 6 exercises | ~30 min |

| Weekend | Active Rest | Light walking (10-15 min) | - | - |

**Key Milestone Week 2:** Patient demonstrates proper breathing technique during resistance exercises. No adverse symptoms with combined training.

---

\### Week 3: Progressive Loading Phase

**Focus:** Increasing aerobic duration, resistance volume progression

| Day | Session Type | Aerobic | Resistance | Total Time |

|-----|-------------|---------|------------|------------|

| Monday | Aerobic | 20 min @ RPE 11-13 | - | ~25 min |

| Tuesday | Combined | 15 min @ RPE 10-12 | 1-2 sets × 12-15 reps, 7 exercises @ RPE 12 | ~35 min |

| Wednesday | Aerobic | 20-22 min @ RPE 11-13 | - | ~27 min |

| Thursday | Rest | - | - | - |

| Friday | Combined | 15 min @ RPE 10-12 | 2 sets × 10-12 reps, 7 exercises | ~35 min |

| Saturday | Aerobic | 15-20 min @ RPE 10-12 | - | ~25 min |

| Sunday | Active Rest | Light walking (15 min) | - | - |

**Key Milestone Week 3:** 4 aerobic sessions achieved. Patient can complete resistance exercises with proper form. Progression to 2 sets on combined days.

---

\### Week 4: Consolidation Phase

**Focus:** Solidifying gains, preparing for independent maintenance

| Day | Session Type | Aerobic | Resistance | Total Time |

|-----|-------------|---------|------------|------------|

| Monday | Aerobic | 25 min @ RPE 11-13 | - | ~30 min |

| Tuesday | Combined | 15 min @ RPE 11-12 | 2 sets × 12-15 reps, 7 exercises @ RPE 12-13 | ~35 min |

| Wednesday | Aerobic | 20-25 min @ RPE 11-13 | - | ~28 min |

| Thursday | Rest | - | - | - |

| Friday | Combined | 15-18 min @ RPE 11-12 | 2 sets × 12-15 reps, 8 exercises @ RPE 12-13 | ~40 min |

| Saturday | Aerobic | 20 min @ RPE 10-12 | - | ~25 min |

| Sunday | Active Rest | Light walking (15-20 min) | - | - |

**End of Program Target:**

- Aerobic: 20-25 minutes continuous at RPE 11-13 (moderate intensity)

- Resistance: 2 sets × 12-15 reps × 8 exercises at RPE 12-13

- Total weekly volume: ~120-150 minutes aerobic exercise

---

\## IV. Condition-Specific Modifications for HFpEF

\### Diastolic Dysfunction Considerations

1. **Avoid High-Intensity Training:** Unlike HFrEF, HFpEF patients may not tolerate high-intensity interval training. The primary limitation is reduced ventricular filling, not pump failure. Exercise should be **continuous, moderate-intensity** rather than interval-based .

2. **Monitor for Exertional Dyspnea:** This is the patient's primary symptom. If dyspnea prevents talking in short sentences, intensity is too high. Reduce workload immediately.

3. **Recognize "Heart Failure Symptoms":**

- Excessive fatigue > 2 hours post-exercise

- New or worsening shortness of breath at rest

- Weight gain > 2 lbs overnight (fluid retention)

- Increased swelling in ankles/feet

- Report any of these to healthcare provider

\### Diabetes Management Considerations

1. **Pre-Exercise Blood Glucose:** Check before exercise. Avoid if < 100 mg/dL (treat with 15g carbs) or > 250 mg/dL with ketones.

2. **Hypoglycemia Risk:** Have fast-acting glucose available during sessions.

3. **Foot Care:** Inspect feet daily; wear appropriate footwear for walking sessions.

\### Hypertension Considerations

1. **Medication Timing:** Beta-blockers may affect HR response. Emphasize RPE over HR targets .

2. **Post-Exercise BP:** Monitor for orthostatic hypotension; cool down adequately.

3. **Hold Exercise for BP:** SBP > 180 or DBP > 110 mmHg.

---

\## V. Safety Monitoring & Emergency Protocols

\### During Each Session (Vital Signs)

| Parameter | Normal Response | Concerning | Action |

|-----------|----------------|------------|--------|

| **Heart Rate** | Gradual rise to target zone | > 120% target or failure to recover | Reduce intensity; if persists, STOP |

| **Blood Pressure (post-exercise)** | Slight decrease or return to baseline | SBP drop > 20 mmHg with symptoms | Assess for orthostatic hypotension |

| **RPE** | 9-13 (training zone) | > 14 (too hard) | Reduce workload immediately |

| **Dyspnea** | Mild, able to speak | Unable to complete sentences | Stop and rest |

| **Oxygen Saturation** | > 95% | < 92% | Stop; reassess; consider medical evaluation |

\### "RED FLAG" Symptoms - STOP EXERCISE IMMEDIATELY

- Chest pain, pressure, tightness (angina equivalent)

- Severe shortness of breath (unable to speak at all)

- Dizziness, lightheadedness, or near-syncope

- Palpitations or irregular heartbeat

- Excessive fatigue disproportionate to effort

- Nausea or diaphoresis

**Emergency Action Plan:** If any red flag symptom develops:

1. Stop exercise immediately

2. Sit patient in a comfortable position

3. Administer oxygen if available

4. Check vital signs

5. Contact supervising physician or emergency services if symptoms do not resolve within 5 minutes of rest

---

\## VI. Summary of FITT-VP Prescription

| Component | Week 1-2 | Week 3-4 | Target Goal |

|-----------|---------|---------|-------------|

| **Frequency (Aerobic)** | 3x/week | 4x/week | 4-5x/week |

| **Intensity (Aerobic)** | RPE 9-11 (40-50% HRR) | RPE 11-13 (50-60% HRR) | RPE 11-13 |

| **Time (Aerobic)** | 10-15 min | 20-25 min | 30-40 min |

| **Type (Aerobic)** | Cycle, walking | Cycle, walking | Variety |

| **Frequency (Resistance)** | 1-2x/week (week 2+) | 2x/week | 2-3x/week |

| **Intensity (Resistance)** | 40-50% 1RM | 50-60% 1RM | 60-70% 1RM |

| **Time/Reps (Resistance)** | 1 set × 10 reps | 2 sets × 12-15 reps | 2-3 sets × 12-15 reps |

| **Type (Resistance)** | Bodyweight + bands | Bands + light weights | Full equipment |

| **Volume** | ~30-40 min/week aerobic | ~90-120 min/week aerobic | 150+ min/week |

---

\## VII. Home Exercise Recommendations (Non-Supervised Days)

**Active Rest Days (2-3 days/week):**

- 10-15 minutes of **walking** at RPE 6-8 (very light)

- Gentle **stretching** (5-10 minutes)

- **Balance exercises**: standing heel-to-toe, single-leg stance with support

**Daily "Movement Snacks" (Break up sedentary time):**

- Standing up every 30-45 minutes

- Walking around the room for 2-3 minutes

- Simple arm and leg movements while seated

---

\## VIII. Patient Education Points

1. **"If it hurts, stop"** - Pain is not a goal in cardiac rehabilitation. RPE 11-13 is "somewhat hard," NOT painful.

2. **"Slow and steady wins the race"** - Progression should be gradual. Increasing duration before intensity is the safest approach.

3. **"Hydrate, hydrate, hydrate"** - Drink water before and after exercise. Monitor fluid balance carefully with HFpEF (discuss fluid limits with physician).

4. **"Track your symptoms"** - Keep a simple daily log of:

- Weight (same time daily)

- Breathing quality

- Exercise session details (duration, RPE)

5. **"Medication consistency"** - Do not adjust heart failure medications without physician consultation, even if "feeling better."

---

\## References

1. Dutch KNGF Guideline on Cardiac Rehabilitation (2023) - ACSM FITT recommendations for heart failure

2. European Society of Cardiology exercise recommendations for NYHA Class 2-3 heart failure

3. CSANZ Position Statement on Exercise and Physical Activity in Cardiac Rehabilitation (2023) - Comprehensive FITT-VP framework

4. American College of Sports Medicine (ACSM) - Exercise guidelines for cardiovascular disease

5. NHS/BHF patient guidance on heart failure exercise safety

6. Physiopedia - Congestive Heart Failure Patient Recommendations

*Disclaimer: This program is designed for supervised cardiac rehabilitation settings. All exercises should be performed under the guidance of qualified healthcare professionals. Individual adjustments may be necessary based on patient response, daily symptoms, and physician recommendations. This program does not substitute for medical advice.*
